# Utilizing Cattle Manure Compost Increases Ammonia Monooxygenase A Gene Expression and Ammonia-oxidizing Activity of Both Bacteria and Archaea in Biofiltration Media for Ammonia Deodorization

**DOI:** 10.1264/jsme2.ME20148

**Published:** 2021-04-27

**Authors:** Rika Kitamura, Toshinori Kozaki, Kazuo Ishii, Masayuki Iigo, Takeshi Kurokura, Kenji Yamane, Isamu Maeda, Kazunori Iwabuchi, Takahiro Saito

**Affiliations:** 1 Center for Bioscience Research and Education, Utsunomiya University, 350 Mine-machi, Utsunomiya-shi, Tochigi-ken 321–8505, Japan; 2 Department of Applied Biological Science, Faculty of Agriculture, Tokyo University of Agriculture and Technology, 3–5–8 Saiwai-cho, Fuchu, Tokyo 183–8509, Japan; 3 Biostatistics Center, Kurume University, 67, Asahi-machi, Kurume, Fukuoka, 830–0011, Japan; 4 Department of Applied Biological Chemistry, Faculty of Agriculture, Utsunomiya University, 350 Mine-machi, Utsunomiya-shi, Tochigi-ken 321–8505, Japan; 5 Department of Agrobiology and Bioresources, Faculty of Agriculture, Utsunomiya University, 350 Mine-machi, Utsunomiya-shi, Tochigi-ken 321–8505, Japan; 6 Department of Bioresource and Environmental Engineering, Faculty of Agriculture, Hokkaido University, 9 Kita 9 jyou nishi, Kita-ku, Sapporo, Hokkaido 060–8589, Japan; 7 Department of Environmental Engineering, Faculty of Agriculture, Utsunomiya University, 350 Mine-machi, Utsunomiya-shi, Tochigi-ken 321–8505, Japan

**Keywords:** biofiltration technology, AOA, AOB, *amoA* gene, microbial community

## Abstract

Malodorous emissions are a crucial and inevitable issue during the decomposition of biological waste and contain a high concentration of ammonia. Biofiltration technology is a feasible, low-cost, energy-saving method that reduces and eliminates malodors without environmental impact. In the present study, we evaluated the effectiveness of compost from cattle manure and food waste as deodorizing media based on their removal of ammonia and the expression of ammonia-oxidizing genes, and identified the bacterial and archaeal communities in these media. Ammonia was removed by cattle manure compost, but not by food waste compost. The next-generation sequencing of 16S ribosomal RNA obtained from cattle manure compost revealed the presence of ammonia-oxidizing bacteria (AOB), including *Cytophagia*, *Alphaproteobacteria*, and *Gammaproteobacteria*, and ammonia-oxidizing archaea (AOA), such as *Thaumarchaeota*. In cattle manure compost, the bacterial and archaeal ammonia monooxygenase A (*amoA*) genes were both up-regulated after exposure to ammonia (fold ratio of 14.2±11.8 after/before), and the bacterial and archaeal communities were more homologous after than before exposure to ammonia, which indicates the adaptation of these communities to ammonia. These results suggest the potential of cattle manure compost as an efficient biological deodorization medium due to the activation of ammonia-oxidizing microbes, such as AOB and AOA, and the up-regulation of their *amoA* genes.

The malodor generated by biodegradation when recycling biological waste into other resources is an issue that must be solved by municipal solid waste management (Smet *et al.*, 1999; Tsai *et al.*, 2008) because it interferes with recycling and comprises a high concentration of ammonia (Nakasaki *et al.*, 1998; Pagans *et al.*, 2006; Hanajima *et al.*, 2010). This issue also needs to be resolved in order to achieve a sustainable, recycling-oriented society that recycles biological resources in discharge areas without the fuel consumption associated with transportation and incineration (Fan *et al.*, 2018). Biofiltration technology is a low-cost and energy-saving method that reduces and eliminates malodors using microbes (Baquerizo *et al.*, 2005; Nanda *et al.*, 2012) without environmental impact (Langenhove *et al.*, 1986; Yasuda *et al.*, 2009; Hort *et al.*, 2013). To promote the survival and proliferation of desirable microbes, it is necessary to provide the conditions required by these microbes because environmental conditions for colonization and growth vary among microbes. Environmental conditions that affect microbial growth include temperature, light (Nakane and Nishizaka, 2017), oxygen content, moisture content, pH, pressure, salt concentrations, chemical component compositions (*e.g.*, minerals) (Lima-Mendez *et al.*, 2015; Kitamura *et al.*, 2016), and microbial interactions (Bollag, J., *et al.* 2002 Impact of soil minerals organic component microorganisms interactions on restoration of terrestrial ecosystems. *17th WCSS* **47**: 1–7) (Huang, 2002; Raghoebarsing *et al.*, 2006; Arashida *et al.*, 2019). Lima-Mendez *et al.* (2015) examined and elucidated the relationships between microbes and chemical components as well as interactions among microbes in a marine environment by data mining. Moisture content and aerobic or anaerobic environmental conditions may be easily adjusted, and temperature and pH naturally change due to microbial activity (Atchley and Clark, 1979). Moreover, microbial interactions and interactions between microbes and chemical components may be essential conditions for the activation of microbes. Therefore, a focus on individual microorganisms and observing how they change in an environment are among the most critical aspects of utilizing microbes.

We have investigated the abilities of ammonia-oxidizing bacteria (AOB) and ammonia-oxidizing archaea (AOA) to deodorize ammonia. Ammonia is deodorized through the nitrification process via oxidation (Kowalchuk and Stephen, 2001). Kitamura *et al.* (2016) compared bacterial community profiles and chemical components, such as pH, electrical conductivity (EC), total nitrogen, phosphorus, potassium, calcium, magnesium, and the carbon/nitrogen ratio, in composts. The findings obtained indicated that chemical components were important factors in the formation of bacterial community profiles based on marked differences in these components in cattle manure compost and food waste compost as well as in the abundance of *Proteobacteria* and AOB in cattle manure compost, which is consistent with the findings of Yamamoto *et al.* (2010) and Hanajima *et al.* (2011). The purpose of the present study was to verify the previously reported conclusion by Kitamura *et al.* (2016), *i.e.*, that cattle manure compost exhibits the ability to remove ammonia. Moreover, we previously compared food waste compost with cattle manure compost to reconsider the recyclability and usability of kitchen refuse generated in urban areas. In the present study, in addition to demonstrating these ammonia removal effects, a comparative analysis was performed between the two compost types.

Therefore, the aim of the present study was to evaluate cattle manure compost and food waste compost as deodorizing media and assess the activities and communities of microbes by (i) measuring the ammonia removal effects of the two compost types after ammonia exposure; (ii) assessing the activities and community structures of microbes associated with the oxidation of ammonia via metagenomic analyses before and after exposure to ammonia using next-generation sequencing of the 16S rRNA region; and (iii) quantifying the relative abundance of bacterial and archaeal *amoA* in both compost types by real-time PCR. The factors needed by bacteria and archaea for ammonia-oxidizing activity were then investigated.

## Materials and Methods

### Compost samples

Two compost types were used—two were produced from dairy cattle manure (MCU and MCK) and two from food waste (FCU and FCN), similar to our previous study (Kitamura *et al.*, 2016).

MCU and MCK were composts of dairy cattle manure collected from the University Farm at Utsunomiya University (Tochigi, Japan; DDD: 36.492669, 139.985245) and the Kurosaki Dairy Farm (Tochigi, Japan; DDD: 36.563623, 140.046150), respectively. FCU and FCN were composts of food waste discarded by the Utsunomiya University Restaurant or from residential homes that were composted at Utsunomiya University or at NPO Eco-clean Jyoso (Ibaraki, Japan; DDD: 35.953012, 139.950235), respectively.

MCU was composted using commercial composting equipment (TECH-550; Tech Corporation). Rice husks (50 kg) were used as a bulking agent in a batch, and 30 kg of dairy cattle manure was added twice a week and agitated once a day. This process was continued for two months, after which the compost was agitated once every few days for two additional months until maturation.

MCK was composted using a scoop wheel fermentation unit (NAZ 60-20 type; Nakamichi Giken). Rice husks (8,000 kg) were used as a bulking agent and mixed with 60,000 kg of dairy cattle manure. The mixture was agitated for one month for fermentation and then dried, after which the compost was matured for 2.5–3 months in piled windrows.

FCU was prepared using the commercial composting equipment used for MCU. Rice husks (50 kg) were used as a bulking agent for one batch, and 15 kg of food waste containing scraps from cooking and leftover food was added. The mixture was agitated once a day, and the addition of food waste was repeated 5 days per week. The composting process was continued for one month, after which the compost was agitated once every few days for two months until maturation.

FCN was composted by a windrow composting facility. Food waste, which was mainly kitchen waste composed of vegetables, was milled with the rotary blade type crush & separator (RB-IItype; Sanyu Kyoritsu Industry), mixed with 0.35% (w/w) quicklime, and added to mature compost as a bulking agent. The mixture was fermented by piling for two weeks, after which the compost was matured by leaving it in windrows for two additional weeks.

### Biofiltration equipment

**Fig. 1.**
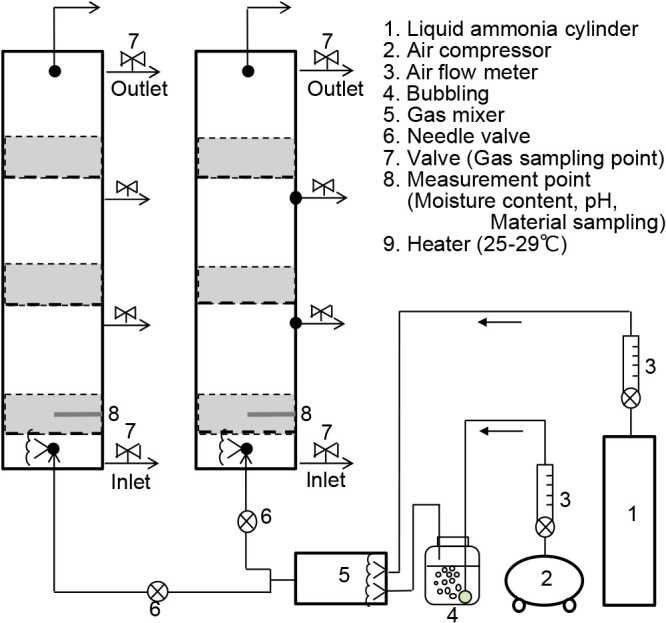
Biofiltration experiment equipment. Biological deodorizing medium was placed at a height of 100‍ ‍mm in the gray-colored areas, and the columns were separated into 3 layers.

The biofiltration equipment used is shown in Fig. 1. Two transparent PVC columns (inner diameter, 147‍ ‍mm) were used and divided into three layers by punching metal plates. They were produced by FUJITA ENGINEERING.

Compressed air was maintained by a compressor and regulator at a rate of 10 L min^–1^ air, and flow was adjusted using a flow meter (RK1250-20-B-1/4-AIR-30L/MIN-0.1MPa-0-D; KOFLOC) and saturated with distilled water by bubbling using an air stone. Distilled water was maintained at 25–29°C with a water heater. Liquid ammonia was adjusted to a rate of 1‍ ‍mL min^–1^ 100% ammonia gas using a flow meter (RK1250-15-SS-1/4-NH3-10mL/MIN-0.1MPa-0-D; KOFLOC.), 1‍ ‍mL min^–1^ 100% ammonia gas and 10 L min^–1^ air (saturated humidity) were mixed, and 100 ppm ammonia gas (saturated humidity) was obtained. Adjusted ammonia gas was branched into two columns by a Y-union and adjusted to 5 L min^–1^ column^–1^ by a needle valve. The empty bed velocity in the columns was 4.6‍ ‍mm s^–1^.

### Ammonia removal experiment

Microbes need water for vigorous activity; therefore, water was added to the manure and food waste composts at approximately 60% wet basis (wb) (Miyatake *et al.*, 2007) and 40% wb (Norose *et al.*, 2009), respectively, which are appropriate moisture contents during composting (Table S2). The reason for the difference in the moisture content between the two compost types is that food waste contains proteins and carbohydrates in the raw material, and when the moisture content exceeds approximately 40% wb, difficulties are associated with maintaining porosity (Norose *et al.*, 2009). An insufficient oxygen supply makes it challenging to activate aerobic microbes; therefore, the two compost types had to have different moisture contents. Water-adjusted composts were maintained at 25°C for 5 days and then packed into the three layers of the column (height of 100‍ ‍mm by an inner diameter of 147‍ ‍mm in each layer) (Baquerizo *et al.*, 2005; Jun and Wenfeng, 2009) for the ammonia removal experiment. Experiments were performed for approximately one month, under which conditions were maintained (empty bed velocity of 4.6‍ ‍mm s^–1^ and inlet ammonia concentration of 100 ppm).

The ammonia gas concentration was measured daily during the first week and subsequently once every 2 days. To sample the gas at the inlet (Fig. 1, bottom valve of No. 7) and outlet (Fig. 1, upper valve of No. 7), the aluminum bag and the sampling nipple of the column were connected using a silicone tube to prevent gas leakage. Gas was collected in the aluminum bag, and the concentration of ammonia was measured using a gas sampler (GV-100S; GASTEC) and gas detector tubes (3M and 3La; GASTEC).

In ammonia removal experiments, MCU samples were replicated 3 times, and the other samples were replicated 2 times. Replicated experiments are labeled as MCU1, MCU2, and MCU3 in the figures and tables, and the same applies to MCK, FCU, and FCN. Regarding MCK1 compost only, the ammonia removal experiment was performed by depositing compost at a height of 100‍ ‍cm because a column without layers was used for this experiment (Table S2). Therefore, it was not possible to accurately compare the pH and microbial communities of MCK1 with those of the other experiments; however, all microbial communities were compared because the experimental materials used for the sequencing analysis were collected 5‍ ‍cm from the bottom of the column (Fig. 1).

### Concentrations of ammonium, nitrite, and nitrate

The moisture content of each material was measured, and 20‍ ‍g of each material was transferred to a bottle containing distilled water (100‍ ‍mL). The bottle was vigorously shaken four times every 15‍ ‍min, and the extraction was performed with 5A filter paper (Advantec MFS). The extraction was diluted 2- to 200-fold at the time of measurement, and the amounts of ammonium, nitrite and nitrate were measured using RQ flex (FUJIWARA SCIENTIFIC). Final concentrations were recalculated using the moisture content and dilution ratio.

### DNA extraction

Small portions collected 5‍ ‍cm from the bottom layer of the column (Fig. 1) using a cylindrical spatula for sampling before and after ammonia removal experiments were packed into 2-mL tubes and stored at –80°C until DNA extraction. Total DNA was extracted from 0.1–0.2‍ ‍g of wet compost samples using the Extrap Soil DNA Kit Plus ver.2 (Nippon Steel & Sumikin Eco-Tech). Extracted DNA was dissolved in 100‍ ‍mL TE buffer and stored at 4°C until PCR amplification.

### PCR amplification and library preparation procedure for eubacterial 16S rRNA

The procedure used is the same as that previously described (Kitamura *et al.*, 2016).

Eubacterial 16S rRNA genes 586 bp in length were amplified using the primers 341F (5′-CCTACGGGAGGCAGCAG-3′) and 907R (5′-CCGTCAATTCCTTTRAGTTT-3′), targeting the V3, V4, and V5 regions, as performed in previous studies (Muyzer *et al.*, 1993; Schmalenberger *et al.*, 2001; Chakravorty *et al.*, 2007). PCR was conducted using the KAPA HiFi HS Ready Mix (KAPA Biosystems) under the following conditions: initial denaturation at 95°C for 3‍ ‍min, 28 cycles of denaturation at 98°C for 30‍ ‍s, annealing at 62°C for 15‍ ‍s, and extension at 72°C for 15‍ ‍s, with a final extension step at 72°C for 3‍ ‍min. PCR products were visualized with SYBR Green I after 1% (w/v) agarose gel electrophoresis at 100‍ ‍V for 25‍ ‍min with 470‍ ‍nm blue light illuminated by a Dark Reader transilluminator (Clare Chemical Research); purified DNA was excised from a gel band by Takara RECOCHIP (Takara Bio).

The purified PCR products of the eubacterial 16S rRNA gene were quantified using the Invitrogen Quant-iT PicoGreen dsDNA Reagent (Life Technologies). Ten nanograms of PCR products at a final concentration of 200 pg mL^–1^ was used for library preparation. DNA libraries were produced using the TruSeq ChIP DNA Sample Prep Kit (Illumina).

### Library preparation procedure for archaeal 16S rRNA

The DNA libraries of the archaeal 16S rRNA gene were produced using the Nextera XT v2 Index Kit (Illumina). The archaeal 16S rRNA gene, 492 bp in length, was amplified using the primers ARCH915 (5′-AGGAATTGGCGGGGGAGCAC-3′) and UNI-b-rev (5′-GACGGGCGGTGTGTRCAA-3′) (Yu *et al.*, 2008). This primer set was previously added to 5′-TCGTCGGCAGCGTCAGATGTGTATAAGAGACAG-3′ [forward primers: ARCH915] and 5′-GTCTCGTGGGCTCGGAGATGTGTATAAGAGACAG-3′ [reverse primers: UNI-b-rev] according to the “16S Metagenomic Sequencing Library Preparation” protocol for the Illumina MiSeq system (Part # 15044223 Rev. B). The first PCR in the protocol was performed using Tks Gflex DNA Polymerase (Takara Bio) under the following conditions: initial denaturation at 94°C for 1‍ ‍min; 35 cycles of denaturation at 98°C for 10‍ ‍s, with annealing at 55°C for 15‍ ‍s and extension at 68°C for 60 s; and a final extension step at 68°C for 5‍ ‍min.

### Verification of lengths and concentrations of libraries

Fragment sizes were verified using a 2100 Bioanalyzer in combination with an Agilent DNA 1000 Kit (both from Agilent Technologies). The concentrations of the libraries were quantified using the KAPA Library Quantification Kit (Kapa Biosystems) and the Thermal Cycler Dice Real Time System (Takara Bio) or LightCycler 96 (Roche Diagnostics). DNA samples were also adjusted to 4 nM using elution buffer (EB).

### Next-generation sequencing analysis

The MiSeq Reagent Kit v2 (500 cycles) or MiSeq Reagent Kit v3 (600 cycles) (Illumina) was used in the next-generation sequencing analysis.

### Classification of sequence reads based on local BLAST searches

Metagenome analysis processes for eubacterial 16S rRNA were performed on a Linux OS (CentOS 6.5 or Scientific Linux 6.5) platform as described by Kitamura *et al.* (2016).

The other metagenome analysis processes were performed using the standard operational procedure of the Mothur program (https://www.mothur.org/wiki/MiSeq_SOP in 2016) (Kozich *et al.*, 2013). The reference alignment was customized based on SILVA_123_SSURef_tax_silva_trunc.fasta in Silva SSU database release 123 (http://www.arb-silva.de/no_cache/download/archive/release_123/).

Only single reads of 250–300 bp with the forward primer were used in the analysis of eubacterial 16S rRNA genes because paired-end reads were not joined. The archaeal 16S rRNA gene was joined with the paired-end reads and used for analyses. Thirty thousand sequences from each library were extracted to classify and count the sequences based on taxonomy. Data from all libraries were compiled using R ver3.1.0 (R core Team, 2015) and used for phylogenetic taxonomy analyses. β diversity was calculated using the QYC distance expressed by Yue and Clayton (Yue and Clayton, 2005). The β values of eubacteria between replicates before exposure to ammonia were cited by Kitamura *et al.* (2016). β diversity is an indicator of the difference between the microbial communities of libraries. A β value of 1 shows that the microbes present in two libraries differ by 100%, and as the β value decreases from 1, the microbes present in two libraries become more similar.

### Relative quantification using real-time PCR

Relative quantification was performed via real-time PCR with a LightCycler 96, and a eubacterial 16S rRNA reference gene and eubacterial *amoA* gene (491 bp) were amplified using the primers 341F and 907R and the primers amoA1F (5′-GGGGTTTCTACTGGTGGT-3′) and amoA2R (5′-CCCCTCKGSAAAGCCTTCTTC-3′) (Yu *et al.*, 2008), respectively. The archaeal 16S rRNA reference gene and archaeal *amoA* gene (539 bp) were amplified using the primers ARCH915 and UNI-b-rev and the primers Arch-amoA98F (5′-TCTAYACHGAYTGGVHDTGGAC-3′) and Arch-amoAR (5′-GCGGCCATCCATCTGTATGT-3′) (Francis *et al.*, 2005), respectively. Real-time PCR was performed using Tks Gflex DNA Polymerase. Fluorescent SYBR Green I Nucleic Acid Stain (Lonza Rockland) was diluted 3,000-fold with distilled water and used in reactions. Real-time PCR conditions were the same as those described in the section “Library preparation procedure for archaeal 16S rRNA”.

### NGS sequencing read accession numbers

The NGS sequencing reads of bacteria before exposure to ammonia (sample name_B) were published by Kitamura *et al.* (2016). The NGS sequencing reads used in the present study have been deposited in the DDBJ Sequence Read Archive with the following accession numbers. Bacterial fastq files after exposure to ammonia are DRX195423 (MCU1_A), DRX195425 (MCU2_A), DRX195424 (MCU3_A), DRX195426 (MCK1_A), DRX195427 (MCK2_A), DRX195428 (FCU1_A), DRX195430 (FCU2_A), DRX195429 (FCN1_A), and DRX195431 (FCN2_A). Archaeal fastq files before exposure to ammonia are DRX195245 (MCU1_B), DRX195247 (MCU2_B), DRX195249 (MCU3_B), DRX195241 (MCK1_B), DRX195243 (MCK2_B), DRX195237 (FCU1_B), DRX195239 (FCU2_B), and DRX195234 (FCN1_B). Archaeal fastq files after exposure to ammonia are DRX195246 (MCU1_A), DRX195248 (MCU2_A), DRX195250 (MCU3_A), DRX195242 (MCK1_A), DRX195244 (MCK2_A), DRX195238 (FCU1_A), DRX195240 (FCU2_A), DRX195235 (FCN1_A), and DRX195236 (FCN2_A).

## Results and Discussion

### Ammonia removal activity

The ammonia removal rate was measured using two types of cattle manure composts (MCU and MCK) and two types of food waste composts (FCU and FCN) as biofilter media.

**Fig. 2.**
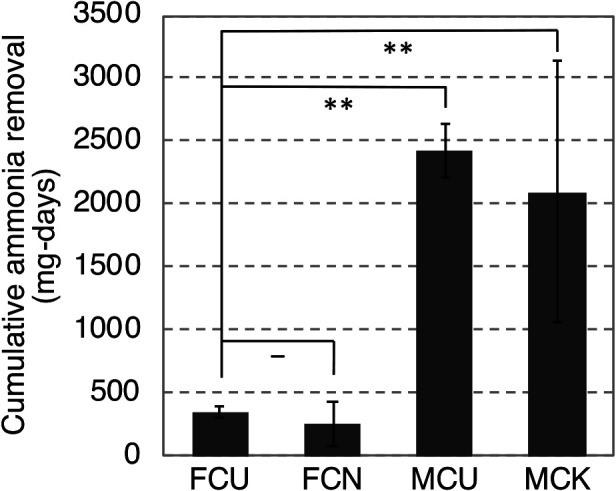
Cumulative ammonia removal amounts by cattle manure composts (MCU, MCK) and food waste composts (FCU, FCN). Significant differences (*P<0.05 and **P<0.01) between ammonia removal effects were observed using Tukey’s HSD test. Columns and error bars represent means±SD (FCU: *n*=2, FCN: *n*=2, MCU: *n*=3, MCK: *n*=2). Tukey’s HSD method was used to evaluate the significance of the results obtained because the number of experimental groups relative to the replicated number indicated that the Bonferroni method was too strict.

The cattle manure composts exerted strong ammonia removal effects (Fig. 2; Fig. S1a). The average amounts of ammonia removed by MCU and MCK were 2.4 and 2.1 g, respectively (Fig. 2), under a total ammonia load of 2.8‍ ‍g for 28 days, and ammonia removal rates were 85.7 and 75%, respectively (Fig. S1a). These values were significantly higher than those achieved by the food waste composts. Nitrite and nitrate were generated by ammonia oxidation in the cattle manure composts (Table 1), which supported the ammonia removal effects exerted by nitrifiers. Concomitantly, pH decreased from the beginning of the experiment (Fig. S2). The pH value of MCU1 finally stabilized at approximately 7.5, which is the optimum pH for nitrifying bacteria and archaea (Yomura *et al.*, 2002; Nicol *et al.*, 2008; Oton *et al.*, 2016). MCK2 did not show an increase in the ammonia removal rate at the beginning of the experiment (Fig. S1a); however, the removal rate increased on approximately day 10, after which it immediately decreased and no further ammonia removal effects were observed. Although a decrease in pH was also noted in MCK, pH remained higher than the optimum pH for nitrifying bacteria. In the food waste composts, ammonia removal was observed on several days in the early stage of the experiment (Fig. 2 and S1b), which was attributed to the adsorption of ammonia into the moist material because the ammonia concentration slightly increased in the moist material at the end of the experiment (Table 1). Additionally, pH did not change. These results are consistent with our previous findings (Kitamura *et al.*, 2016), indicating that the removal of ammonia was possible with cattle manure compost, but not with food waste compost.

### Accumulation of ammonia, nitrite, and nitrate

In MCU, the accumulation of ammonium was observed in the lower and middle layers at the same concentration, the accumulation of nitrite was only detected in the lower layer, and the accumulation of nitrate was noted in the lower, middle, and upper layers in that order (Table 1). On the other hand, in MCK2, the accumulation of all substances was observed in all layers at approximately the same concentrations (Table 1). The bulk density of MCK2 was approximately half that of MCU (Table S2), suggesting that only the lower and middle layers were not sufficiently treated, and accumulation also occurred in the upper layer. The cumulative amounts of each substance per unit volume of soil moisture in MCK2 were almost equal in all layers, and these compounds appeared to be saturated (Table 1). Therefore, the reason why the removal effect in MCK2 did not continue may have been because accumulated nitrite and nitrate inhibited nitrification.

It is important to maintain denitrification in order to sustain the function of a biofilter. Yasuda *et al.* (2019) suggested nitrogen removal by the addition of thiosulfate. Further studies are needed on the conditions that prevent nitrogen accumulation and on the microbes contributing to denitrification.

### Bacterial and archaeal community variations

**Fig. 3.**
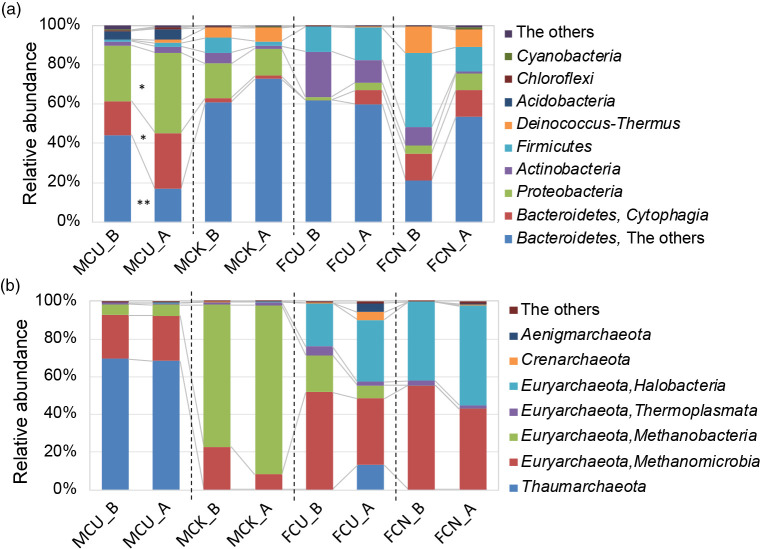
(a) Bacterial and (b) archaeal community profiles at the phylum level, as classified using the Silva SSU database, and relative abundance before (sample name_B) and after (sample name_A) exposure to ammonia. Significant differences (*P<0.05 and **P<0.01) before and after ammonia exposure were observed using paired *t*-tests. Columns represent the means of replicates (MCU: *n*=3, MCK: *n*=2, FCU: *n*=2, FCN: *n*=2). Relative abundance before exposure to ammonia (sample name_B) was reproduced from Kitamura *et al.* (2016).

In the phylum level classification, the MCU and MCK libraries contained the same phyla and had similar bacterial and archaeal community profiles (Fig. 3 and S3), but different bacterial and archaeal species (Tables S3 and S4).

In MCU, which exerted strong deodorizing effects, the presence of both nitrifying bacteria and archaea was confirmed (Tables S3 and S4). Based on the relative abundance of MCU bacterial taxonomic groups, the growth rate of putative*Bacteroidetes-Cytophagia* and putative *Proteobacteria* after exposure to ammonia showed a significant difference at <0.05% (Fig. 3a). In the MCU bacterial community, putative *Cytophagia-Flexibacter sp.* (JQ337769, JQ337592), putative *Alphaproteobacteria* (HQ912787), and putative *Gammaproteobacteria* (ADGO01079279), which include nitrifying bacteria, increased after exposure to ammonia (Table S3). The identified sequences (JQ337769, JQ337592, HQ912787, and ADGO01079279) were previously detected in cattle manure compost (Allgaier *et al.*, 2010; Tian *et al.*, 2013). *Flexibacter canadensis* uses ammonia as a nitrogen source and reduces nitrate (Christensen, 1980), while *F. ruber* uses nitrate ions as a nitrogen source and reduces nitrate (Lewin, 1969; Nakagawa *et al.*, 2002). Among archaea, putative *Thaumarchaeota* of nitrifying archaea predominated and accounted for approximately 74–80% of the community both before and after exposure to ammonia (Table S4); however, no increase or decrease in each archaeal group was observed after exposure to ammonia. Previous studies reported that the phylum *Thaumarchaeota* plays a large role in the oxidation of ammonia (Könneke *et al.*, 2005; De La Torre *et al.*, 2008; Hatzenpichler *et al.*, 2008; Blainey *et al.*, 2011); however, Hatzenpichler *et al.* (2008) and Pratscher *et al.* (2011) indicated that soil AOA were observed when total ammonia concentrations were low (15‍ ‍μg NH_4_^+^ N [g dw soil]^–1^), while AOB responded to high ammonia concentrations (>100‍ ‍μg NH_4_^+^ N [g dw soil]^–1^). The total ammonium content was 0.13‍ ‍g (Table S2) before exposure to ammonia, and the solid phase content was 1.4‍ ‍kg (dw solid) (Table 1) in MCU1; the initial ammonium nitrogen content was calculated to be 72.1‍ ‍μg NH_4_^+^ N (g dw solid)^–1^. In this experiment, when 100 ppm ammonia gas was aerated, the ammonium nitrogen content exceeded 100‍ ‍μg NH_4_^+^ N (g dw solid)^–1^, suggesting that eubacteria were dominant. Moreover, the majority of ammonia-oxidizing archaea belong to groups I.1a (DeLong, 1992) and I.1b (Bintrim *et al.*, 1997; Ochsenreiter *et al.*, 2003) of the phylum* Thaumarchaeota*. Putative *Thaumarchaeota* were detected in MCU and identified in cattle manure compost by Yamamoto *et al.* (2011), and may belong to the I.1b group.

The other cattle manure compost, MCK, exerted weak ammonia removal effects, and nitrifying bacteria and archaea were not sufficiently detected. Significant changes in the phylum classification were not observed for MCK (Fig. S3). In terms of the abundance ratio of each bacterial species, putative *Flavobacteria-Galbibacter* (EU928746) significantly increased and was 100% homologous to *G. marinus* strain ck-I2-15, which is a reported denitrifier (Table S3) (Khan *et al.*, 2007; Li *et al.*, 2013). However, this strain may have functioned as a nitrifier rather than a denitrifier in the present study because nitrate and nitrite accumulated after exposure to ammonia (Table 1), indicating that denitrification did not occur. Putative *Gammaproteobacteria* (JN256105), which include nitrifying bacteria, slightly increased after exposure to ammonia; however, the abundance ratio was low (Fig. S3a and Table S3). Nitrifying archaea were not sufficiently detected in MCK.

The food waste composts, FCU and FCN, did not exert ammonia removal effects, and a metagenome analysis using 16S rRNA sequences indicated different increases/decreases in the abundance of bacterial and archaeal species between experiments 1 and 2 (Tables S3 and S4). The reason for these results may be that the microorganisms were either not affected or unfavorably affected by ammonia. These microorganisms appeared to have caused inconsistent phenomena after exposure to ammonia either because they were killed by ammonia or they were present in different locations.

### Variations in β values

The maximum β diversity value of 1 indicates completely different microbial communities, while smaller values indicate similar microbial communities and the presence of the same microbes.

In the cattle manure compost MCU, the β diversity values for eubacteria between replicates changed from 0.46–0.57 before exposure to ammonia to 0.07–0.16 after exposure (Table 2), which indicated that 84–93% of the bacteria were ultimately the same. In MCK, the value changed from 0.94 before exposure to ammonia to 0.55 after exposure (Table 2), and 45% of the bacteria were the same. These results suggest that the bacterial community in cattle manure compost adapted to a specific environment containing ammonia through the presence of microorganisms that grow, decrease, or die when exposed to ammonia. Similar results were obtained for archaea (Tables 3 and S5). The β diversity values for eubacteria before exposure to ammonia were previously reported (Kitamura *et al.*, 2016). Regarding food waste compost, changes in β diversity values before and after exposure to ammonia for eubacteria and archaea were disparate, and many different species were observed in each replicate (Tables 2, 3, and S5); therefore, the bacterial and archaeal species in food waste composts were considered to be incapable of adapting to ammonia.

These results indicated that bacteria and archaea with ammonia-assimilation abilities increased after exposure to ammonia, while ammonia-sensitive bacteria and archaea decreased.

### Variations in *amoA* gene abundance

**Fig. 4.**
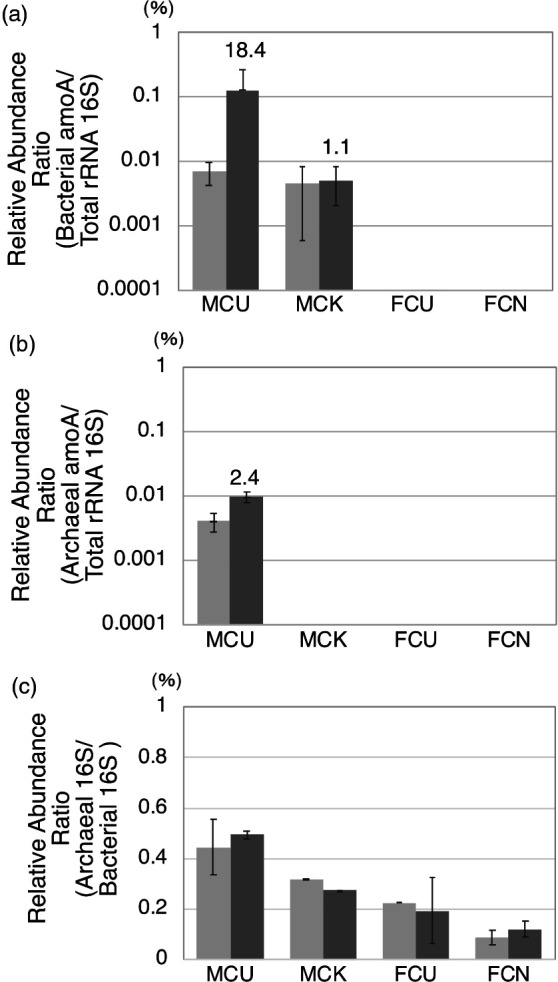
Changes in the abundance ratio of (a) the bacterial amoA gene normalized based on total bacterial and archaeal 16S rRNA genes, (b) the archaeal amoA gene normalized based on total bacterial and archaeal 16S rRNA genes, and (c) the archaeal 16S rRNA gene normalized based on the bacterial 16S rRNA gene before (left column) and after (right column) exposure to ammonia using real-time PCR. Columns and error bars represent means±SD (MCU: *n*=3, MCK: *n*=2, FCU: *n*=2, FCN: *n*=2). The numbers at the top of the right column represent the before/after fold ratio.

In MCU, bacterial and archaeal *amoA* genes both increased after ammonia exposure (Fig. 4 and S4). The ratios of the bacterial *amoA* gene to the total amount of bacterial and archaeal 16S rRNA were 0.004–0.01% before and 0.024–0.32% after exposure to ammonia (Fig. S4a); therefore, the average fold ratio was 18.4 (Fig. 4a). The ratios of the archaeal *amoA* gene to the total amounts of bacterial and archaeal 16S rRNA were 0.002–0.005% before and 0.007–0.012% after exposure to ammonia (Fig. S4b); therefore, the average fold ratio was 2.4 (Fig. 4b). The ratio of archaeal 16S rRNA to bacterial 16S rRNA was approximately 0.4% in cattle manure composts (Fig. 4c). The amount of the bacterial *amoA* gene was similar to that of the archaeal *amoA* gene before exposure to ammonia, while that of the bacterial *amoA* gene was 3- to 20-fold higher than that of the archaeal *amoA* gene after exposure to ammonia. Although putative *Thaumarchaeota* in MCU are nitrifiers, qPCR results did not show any significant increase or decrease after exposure to ammonia, similar to the results of the metagenomic analysis. These results suggest that eubacteria and not archaea were responsible for the removal of ammonia in the present study. In MCK, the amount of the bacterial *amoA* gene slightly changed before and after exposure to ammonia (Fig. 4a), and the archaeal *amoA* gene was not detected (Fig. 4b).

The abundance ratios of the *amoA* gene by quantitative PCR were less than those obtained for AOB and AOA in the phylogenetic classification elucidated by the MiSeq sequencing analysis. Therefore, the differences observed in abundance ratios according to the analysis methods may have been caused by insufficient PCR efficiency, not analyzing the *amoB* gene and *amoC* gene, or other factors. For example, there may be other genes that remove ammonia besides *amoA*, *amoB*, and *amoC*. Even bacteria and archaea that are homologous to AOB and AOA according to the 16S rRNA gene analysis may have different sequences other than the 16S rRNA gene because the 16S rRNA gene is highly conserved (Chakravorty *et al.*, 2007). Therefore, a corresponding analysis and evaluation are necessary in the future.

The adaptation of eubacterial and archaeal species to ammonia may be assessed by clarifying whether the species increases or decreases when exposed to ammonia. The present results indicated that putative nitrifying bacteria were more involved in ammonia oxidation than putative nitrifying archaea. AOA are predominantly abundant in oligotrophic environments (Hatzenpichler *et al.*, 2008; Gubry-Rangin *et al.*, 2011), and AOB in eutrophic environments (Di *et al.*, 2009; Jia and Conrad, 2009; Martens-Habbena *et al.*, 2009; Koper *et al.*, 2010). Compost is generally a eutrophic environment because it contains a large amount of nitrogen. The group I.1b *Thaumarchaeota* of nitrifying archaea was previously reported to be abundant in neutral-alkalinophilic pH soils (Gubry-Rangin *et al.*, 2010; Oton *et al.*, 2016), and some species inhabit eutrophic environments (Oton *et al.*, 2016). Furthermore, soil AOA were observed when total ammonia concentrations were less than 15‍ ‍μg NH_4_^+^ N (g dw soil)^–1^, while AOB responded to high ammonia concentrations (>100‍ ‍μg NH_4_^+^ N [g dw soil]^–1^) (Hatzenpichler *et al.*, 2008; Pratscher *et al.*, 2011). In the present study, 100 ppm of ammonia gas was used, and the conversion of ammonia in liquid that accumulated in the compost to a dry basis of the solid showed that this concentration exceeded 100‍ ‍μg NH_4_^+^ N (g dw solid)^–1^ (Table 1). Since compost is an alkaline medium containing a large amount of nitrogen, and based on the ammonia concentration in the compost, eubacteria were considered to be more responsible for nitrogen circulation, such as ammonia oxidation, than archaea.

Therefore, AOB and AOA were present in MCU, which exerted strong ammonia removal effects, and bacterial *amoA* also increased. Although the function of AOA currently remains unclear, it is likely that microbial interactions often activate their function. Microbial interactions are also an important research subject for the future.

## Conclusions

In the present study, we demonstrated, for the first time, that cattle manure compost exerted ammonia removal effects and these effects were enhanced in cattle manure compost containing both AOB and AOA. In cattle manure compost exerting strong ammonia removal effects, the abundance of *Cytophagia* and *Proteobacteria* in the bacterial community increased after exposure to ammonia, and *Thaumarchaeota* accounted for more than 75% of the archaeal communities throughout the experiment. Bacterial *amoA* genes coexisting with archaeal *amoA* genes increased after exposure to ammonia, and the bacterial and archaeal communities between the replicates were more homologous and consistent after than before exposure to ammonia. The presence of both AOB and AOA and their microbial interactions appear to be required for the ammonia-oxidizing effects that contribute to their colonization and growth.

## Citation

Kitamura, R., Kozaki, T., Ishii, K., Iigo, M., Kurokura, T., Yamane, K., et al. (2021) Utilizing Cattle Manure Compost Increases Ammonia Monooxygenase A Gene Expression and Ammonia-oxidizing Activity of Both Bacteria and Archaea in Biofiltration Media for Ammonia Deodorization. *Microbes Environ ***36**: ME20148.

https://doi.org/10.1264/jsme2.ME20148

## Supplementary Material

Supplementary Material

## Figures and Tables

**Table 1.** Cumulative amount of ammonium, nitrite, and nitrate in cattle manure composts and food waste composts before and after exposure to ammonia in biofiltration media.

		MCU1			MCU2			MCU3			MCK2			FCU2			FCN1			FCN2	
		Before	After		Before	After		Before	After		Before	After		Before	After		Before	After		Before	After
Moisture content (%, wb)	Upper	60.3	58.0		56.6	55.4		59.0	58.9		58.3	56.6		58.5	58.3		37.1	38.9		34.7	35.0
	Middle		57.1			55.4			59.3			55.8			59.7			37.4			35.7
	Lower		60.3			55.4			58.2			55.4			59.7			40.7			37.8
Liquid phase volume (L)	Upper	2.13	0.65		1.52	0.49		1.64	0.54		1.01	0.31		2.05	0.70		1.38	0.50		1.44	0.48
	Middle		0.62			0.49			0.55			0.30			0.74			0.47			0.50
	Lower		0.71			0.49			0.53			0.30			0.74			0.53			0.55
Ammonium	Upper (g L^–1^)	0.06	0.38		0.14	0.79		0.23	0.11		0.49	3.42		2.30	3.93		1.43	2.33		1.53	2.06
	Middle (g L^–1^)		5.46			4.03			4.51			3.98			3.20			2.42			2.39
	Lower (g L^–1^)		7.68			4.03			4.59			3.74			3.19			2.24			1.74
	Total amount (g)	0.13	9.09		0.21	4.30		0.38	4.97		0.49	3.36		4.71	7.45		1.97	3.48		2.20	3.14
Nitrite	Upper (g L^–1^)	0.32	0.14		0.20	0.01		0.09	0.02		0.27	15.44		0.08	0.14		0.20	0.22		0.28	0.09
	Middle (g L^–1^)		2.56			0.12			0.85			16.73			0.13			0.26			0.09
	Lower (g L^–1^)		7.99			9.32			13.24			15.44			0.12			0.27			0.08
	Total amount (g)	0.67	7.35		0.30	4.59		0.14	7.47		0.27	14.38		0.16	0.28		0.28	0.37		0.40	0.13
Nitrate	Upper (g L^–1^)	5.00	5.20		5.09	18.41		4.27	10.30		3.90	93.13		0.38	0.70		Lo	Lo		0.57	0.43
	Middle (g L^–1^)		41.80			31.46			34.01			102.45			0.52			Lo			0.50
	Lower (g L^–1^)		84.60			43.51			94.45			91.88			0.51			Lo			0.33
	Total amount (g)	10.64	89.37		7.75	45.38		7.00	74.25		3.93	86.85		0.78	1.25		Lo	Lo		0.82	0.64

**Table 2.** Summary of β diversities between putative bacterial communities after exposure to ammonia based on a DNA sequence similarity search within the Silva SSU database.

	MCU1	MCU2	MCU3	MCK1	MCK2	FCU1	FCU2	FCN1
MCU1								
MCU2	**0.13839**							
MCU3	**0.07302**	**0.16488**						
MCK1	0.97049	0.93216	0.98483					
MCK2	0.99914	0.99950	0.99887	**0.54878**				
FCU1	0.99998	0.99998	0.99999	0.99983	0.99858			
FCU2	0.99938	0.99908	0.99983	0.99454	0.99726	**0.81235**		
FCN1	0.99974	0.99917	0.99976	0.97949	0.98044	0.99920	0.98438	
FCN2	0.99974	0.99918	0.99968	0.99752	0.99878	0.99833	0.99397	**0.69116**

Notable numbers are written in bold letters.

**Table 3.** Summary of β diversities between putative archaeal communities after exposure to ammonia based on a DNA sequence similarity search within the Silva SSU database.

	MCU1	MCU2	MCU3	MCK1	MCK2	FCU1	FCU2	FCN1
MCU1								
MCU2	**0.00048**							
MCU3	**0.00077**	**0.00007**						
MCK1	0.97660	0.97520	0.97441					
MCK2	0.98076	0.98025	0.97945	**0.41148**				
FCU1	0.72207	0.71190	0.70760	0.92419	0.96735			
FCU2	0.92988	0.92907	0.92645	0.95825	0.98454	**0.83122**		
FCN1	0.99840	0.99867	0.99833	0.99695	0.99645	0.99677	0.99943	
FCN2	0.99962	0.99977	0.99965	0.99867	0.99811	0.99999	0.99989	**0.39315**

Notable numbers are written in bold letters.
