# Supplementary material for: Utilizing Cattle Manure Compost Increases Ammonia Monooxygenase A Gene Expression and Ammonia-oxidizing Activity of Both Bacteria and Archaea in Biofiltration Media for Ammonia Deodorization
Source: Microbes Environ. 2021 Apr 27;36(2):ME20148. doi: 10.1264/jsme2.ME20148 (PMC8209447; doi:10.1264/jsme2.ME20148)
Supplement: Supplementary file 1 — Supplementary Material [file 36_20148_s1.pdf]

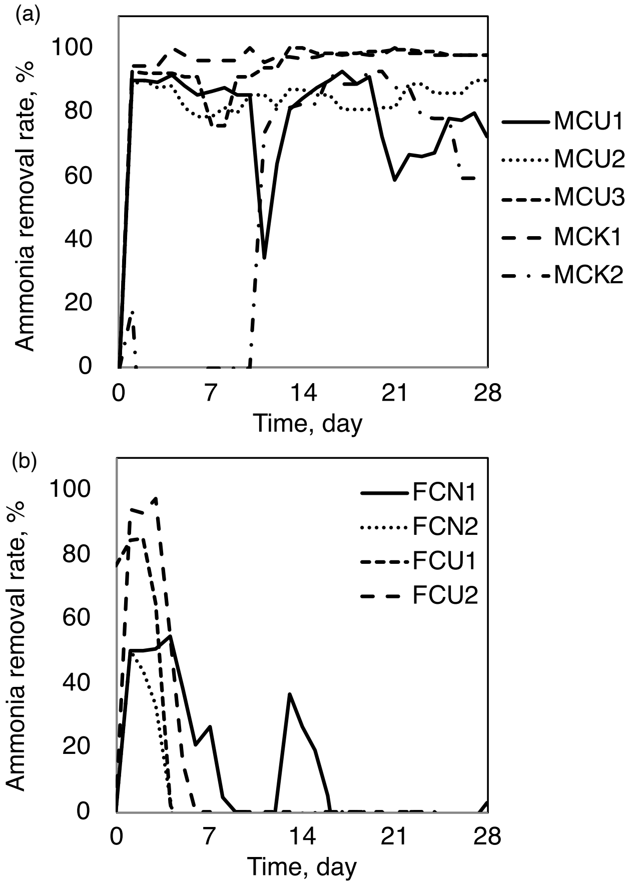

Fig. S1 Temporal variation of the ammonia removal rate in the (a) cattle manure compost and (b) food waste compost.

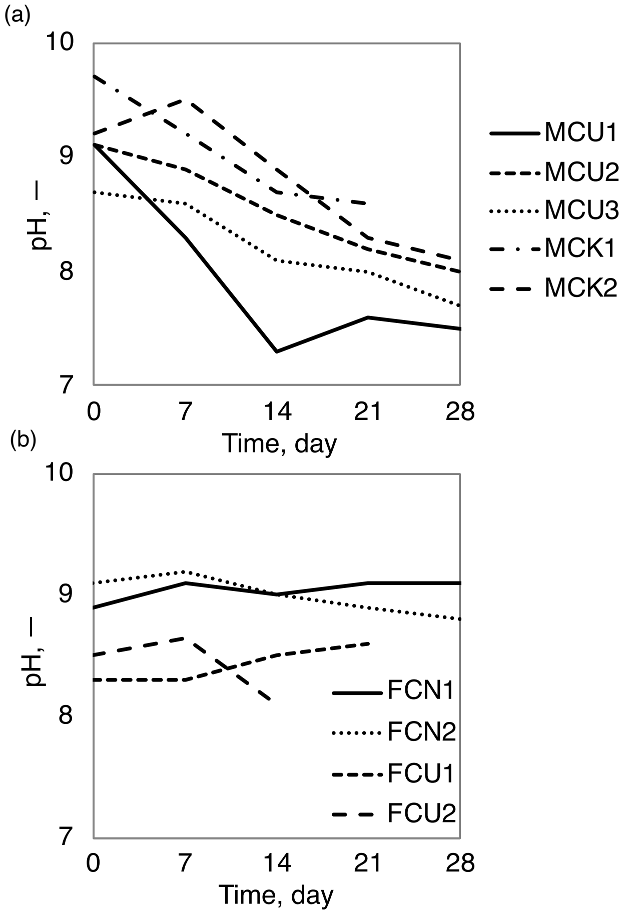

Fig. S2 Temporal variation of the pH in the (a) cattle manure compost and (a) food waste compost.

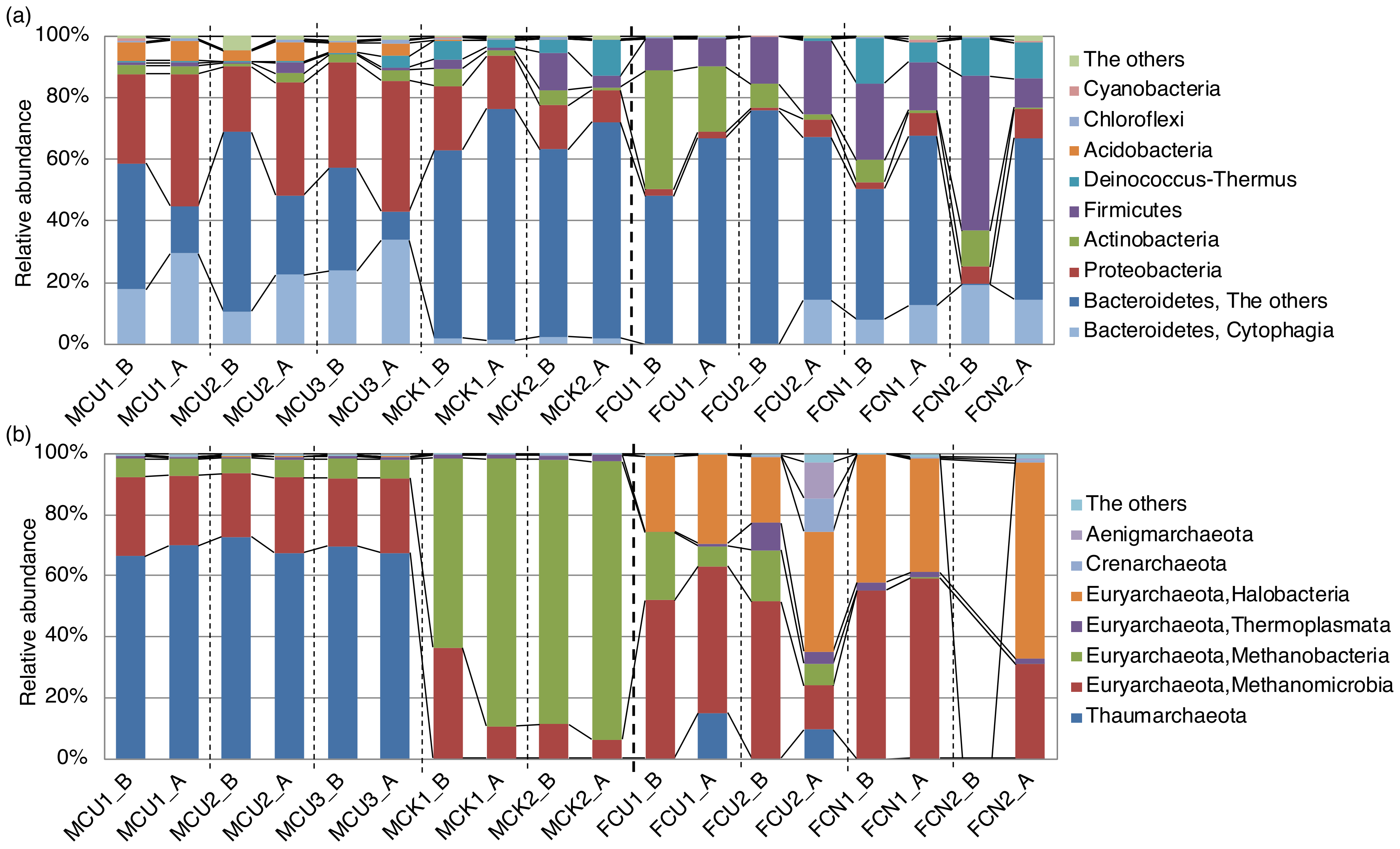

Fig. S3 (a) Bacterial and (b) archaeal community profiles representing all replicates (MCU: n=3, MCK: n=2, FCU: n=2, FCN: n=2) at the phylum level before (sample name_B) and after (sample name_A) ammonia exposure, as classified using the Silva SSU database. The relative abundances before ammonia exposure (sample name_B) are reproduced from Kitamura *et al.* (2016).

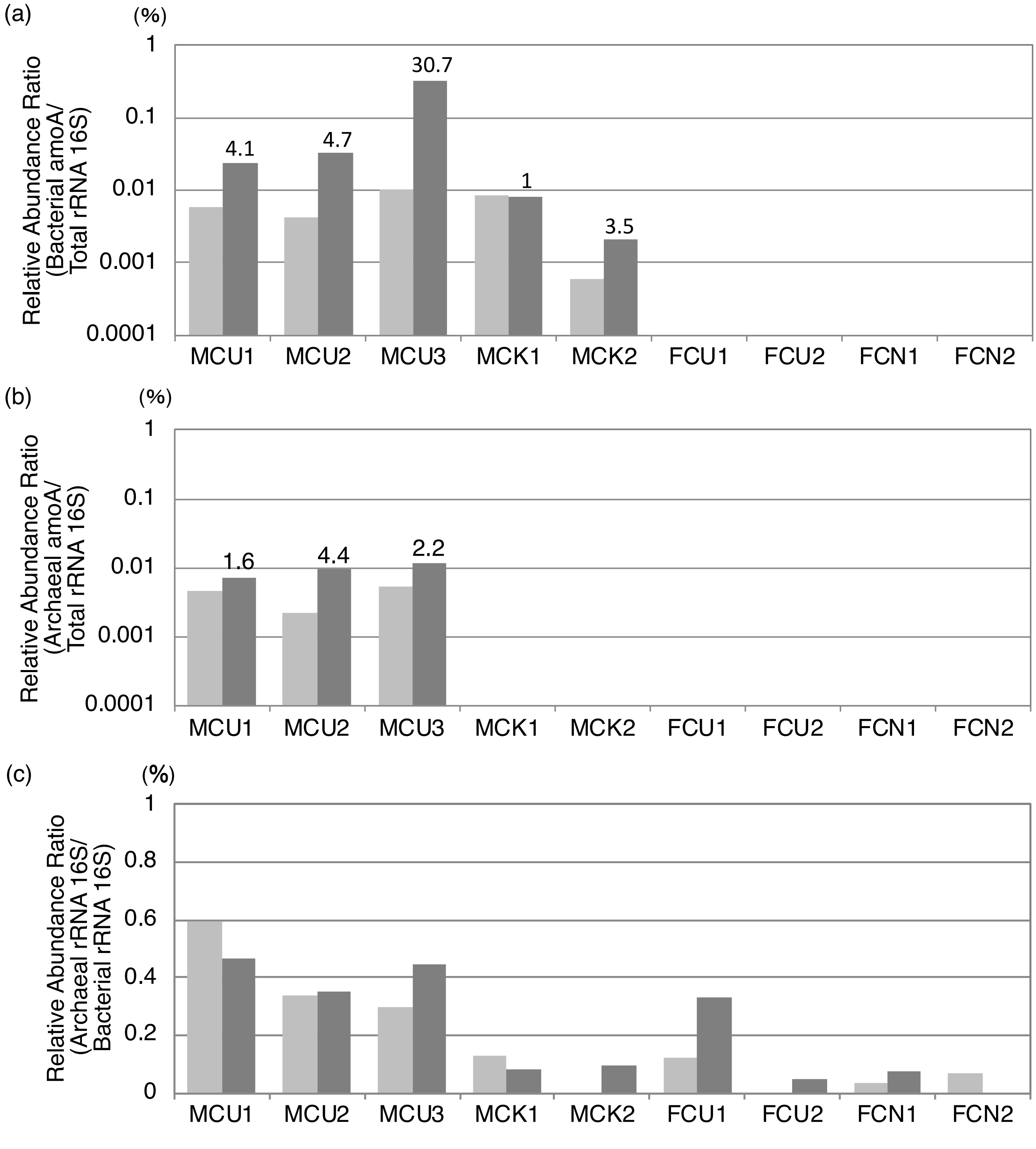

Fig. S4 Relative quantification representing all replicates (MCU: n=3, MCK: n=2, FCU: n=2, FCN: n=2) of (a) the bacterial amoA gene normalized based on the total bacterial and archaeal 16S rRNA genes, (b) the archaeal amoA gene normalized based on the total bacterial and archaeal 16S rRNA genes, and (c) the archaeal 16S rRNA gene normalized with a bacterial 16S rRNA gene before (left column) and after (right column) ammonia exposure using real-time PCR. The numbers at the top of the color bar represent the before/after fold ratio.

Table S1 Properties of the mature composts.

	MCU	MCK	FCU	FCN
Moisture content (%, wet weight)	41.1	20.0	14.6	19.0
pH	9.1	9.9	8.0	8.8
Volatile matter content (%, dry weight)*1	67.7	71.5	70.8	37.9
Real density (g/cm^3)*2	1.66	1.65	1.73	2.05

*1: The volatile matter is the material containing carbon that is converted to gas by combustion, i.e., the volatile matter content is the difference between the dry weight of the compost before combustion and the weight of the ash yield after combustion expressed as a percentage of the initial weight.
*2: The real density is the solid weight per unit volume of solids, not including the gas and liquid phase.

Table S2 Physical characteristics of the biofiltration media.

	MCU1	MCU2	MCU3	MCK1*1	MCK2	FCU1	FCU2	FCN1	FCN2
Filled material weight (kg)	3.53	2.69	2.78	4.86[1]	1.73	2.33	3.50	3.72	4.14
Bulk density*2 (kg/m3)	695.0	805.0	554.0	318.5	327.0	458.5	710	727.5	535.0
Moisture content (%, wet weight)	60.3	56.6	59.0	50.3	58.3	38.0	58.5	37.1	34.7
Solid phase volume*3 (m^3)	0.88×10^{-3}	0.72×10^{-3}	0.65×10^{-3}	1.52×10^{-3}	0.42×10^{-3}	0.83×10^{-3}	0.83×10^{-3}	1.23×10^{-3}	1.23×10^{-3}
Liquid phase rate*4 (%, vol.)	41.7	29.9	32.2	16.0	19.8	17.4	40.1	27.1	28.2
Solid phase rate*4 (%,vol.)	17.3	14.0	12.8	9.9	8.3	16.3	16.2	24.2	24.2
Gas phase rate *4 (%.vol.)	41.0	56.1	55.1	74.1	71.9	66.3	43.7	48.7	47.6
Ammonia loading mass (µg/g dw solid/day)	357.2	428.8	439.2	207.3	693.9	346.5	344.7	213.9	185.2

*1: The experimental conditions were different from those of the other experiments, and the thickness of the medium was 1000 mm instead of 100 mm × 3 layers.
*2: The bulk density is the material weight including moisture per unit volume, including solid, liquid and gas phases.
*3: The solid phase volume is the volume of the solid phase calculated from the filled material weight, the moisture content and the real density.
*4: Each value of the three phase rates is the ratio of that phase to the total for all three phases.

Table S3 Variations in the putative bacterial species and abundance ratios for the top three at points before and after ammonia exposure.

	Relative abundance ratio (%)						Most Related Sequence		
	Experiment 1		Experiment 2		Experiment 3				
	Before	After	Before	After	Before	After	Similarity(%)	Accession number	Phylum, Class, Family or genus
MCU	27.1	2.0	20.1	4.9	8.4	1.2	100	FJ675178	Bacteroidetes, Flavobacteria, Flavobacterium
	0.0	0.0	23.6	3.7	5.0	0.8	97	LN561785	Bacteroidetes, Flavobacteria, Flavobacterium
	12.1	19.4	6.9	16.4	16.5	22.3	99	JQ337769	Bacteroidetes, Cytophagia, Flexibacter
	4.5	7.9	1.9	4.5	4.9	7.9	99	JQ337592	Bacteroidetes, Cytophagia, Flexibacter
	9.0	13.0	5.5	11.6	14.4	17.0	100	HQ912787	Proteobacteria, Alphaproteobacteria, Altererythrobacter
	4.0	5.6	2.6	6.5	2.7	4.3	100	ADGO 01079279	Proteobacteria, Gammaproteobacteria, Miscellaneous
MCK	25.2	23.4	0.0	0.0			100	FJ675178	Bacteroidetes, Flavobacteria, Flavobacterium
	7.7	3.6	0.3	0.1			100	FJ674881	Bacteroidetes, Flavobacteria, Flavobacterium
	6.5	6.6	0.0	0.0			100	JQ337602	Bacteroidetes, Flavobacteria, Flavobacterium
	2.2	26.3	32.7	63.5			100	EU928746	Bacteroidetes, Flavobacteria, Galbibacter
	3.8	2.1	8.2	0.1			100	JQ337604	Bacteroidetes, Sphingobacteriia, Pedobacter
	0.7	0.4	1.0	4.5			99	HQ727582	Deinococcus-Thermus, Deinococci, Truepera
	6.3	7.1	1.0	2.3			100	JN256105	Proteobacteria, Gammaproteobacteria, uncultured Xanthomonadaceae
	0.3	0.0	3.6	1.3			100	X76443	Firmicutes, Bacilli, Bacillus
FCU	12.3	14.4	3.4	0.3			99	GQ136831	Bacteroidetes, Flavobacteria, Ulvibacter
	19.7	28.0	57.7	8.4			99	AM982677	Bacteroidetes, Sphingobacteriia, uncultured Sphingobacteriaceae
	11.9	15.8	13.1	1.9			100	HM251788	Bacteroidetes, Sphingobacteriia, uncultured Sphingobacteriaceae
	0.0	0.1	0.0	15.7			96	FJ380161	Bacteroidetes, Sphingobacteriia, uncultured Saprospiraceae
	0.0	0.0	0.0	9.4			98	GQ263495	Bacteroidetes, Sphingobacteriia, uncultured Chitinophagaceae
	23.8	11.8	1.5	0.2			100	X91032	Actinobacteria, Actinobacteria, Brachybacterium
	0.0	1.7	6.3	0.8			100	HQ716171	Firmicutes, Bacilli, Atopostipes
	0.0	0.0	0.0	9.7			99	JF894318	Firmicutes, Bacilli, Paenibacillus
FCN	40.6	8.5	0.0	33.6			99	NR_108506	Bacteroidetes, Flavobacteria, Psychroflexus
	1.6	20.1	0.1	4.6			100	EU369141	Bacteroidetes, Flavobacteria, Salinimicrobium
	0.0	4.9	0.1	0.0			100	JF979273	Bacteroidetes, Flavobacteria, Salinimicrobium
	0.0	1.0	0.0	6.4			95	JX391813	Bacteroidetes, Flavobacteria, Owenweeksia
	6.1	1.4	15.8	3.2			98	JQ923476	Bacteroidetes, Sphingobacteriia, Aliifodinibius
	13.6	1.6	10.8	7.7			95	JQ978864	Deinococcus-Thermus, Deinococci, Truepera
	5.0	1.6	10.4	2.6			100	DQ129366	Firmicutes, Bacilli, Bacillus

Table S4 Variations in the putative archaeal species and abundance ratios for the top three at points before and after ammonia exposure.

	Relative abundance ratio (%)						Most Related Sequence		
	Experiment 1		Experiment 2		Experiment 3				
	Before	After	Before	After	Before	After	Similarity(%)	Accession number	Phylum, Class
MCU	74.0	75.3	79.5	75.1	77.8	74.6	100	AB541693	Thaumarchaeota, unclassified
	19.7	17.1	15.1	18.7	16.4	19.1	100	AB541590	Euryarchaeota, Methanomicrobia
	1.8	1.8	1.4	1.8	2.0	1.9	100	GQ214434	Euryarchaeota, Methanobacteria
MCK	36.1	8.0	9.5	3.6	−	−	100	AB541590	Euryarchaeota, Methanomicrobia
	27.0	41.6	41.5	14.3	−	−	100	AB104858	Euryarchaeota, Methanobacteria
	15.9	22.6	20.4	42.5	−	−	100	GQ214434	Euryarchaeota, Methanobacteria
	4.9	7.2	6.2	16.2	−	−	100	KF697734	Euryarchaeota, Methanobacteria
FCU	0.0	13.8	0.0	4.3	−	−	100	AB541693	Thaumarchaeota, unclassified
	12.9	35.6	26.4	4.5	−	−	100	AB541590	Euryarchaeota, Methanomicrobia
	9.6	2.1	1.0	0.1	−	−	73	JX192605	unclassified, unclassified
	9.0	5.6	6.9	1.3	−	−	73	FN994974	unclassified, unclassified
	0.0	0.0	10.1	0.0	−	−	100	GQ214434	Euryarchaeota, Methanobacteria
	0.0	0.0	0.0	3.9	−	−	75	AJ347789	unclassified, unclassified
FCN	58.6	64.2	−	31.4	−	−	99	AB236082	Euryarchaeota, Methanomicrobia
	33.0	24.9	−	59.3	−	−	100	AB663469	Euryarchaeota, Halobacteria
	3.2	4.6	−	1.7	−	−	100	AF251941	Euryarchaeota, Halobacteria
	2.3	1.8	−	1.7	−	−	98	GQ365371	Euryarchaeota, Thermoplasmata

Table S5 Summary of the β-diversities between the putative archaeal communities before ammonia exposure determined based on a DNA sequence similarity search within the Silva SSU database in a previous report (Kitamura *et al*.,2016)

	MCU1	MCU2	MCU3	MCK1	MCK2	FCU1	FCU2	FCN1
MCU1								
MCU2	**0.00827**							
MCU3	**0.00414**	**0.00079**						
MCK1	0.89939	0.93067	0.92096					
MCK2	0.97017	0.97892	0.97519	**0.33545**				
FCU1	0.95881	0.97183	0.96824	0.80967	0.95635			
FCU2	0.91417	0.94174	0.93329	0.48953	0.83283	**0.50422**		
FCN1	0.99927	0.99916	0.99916	0.99260	0.99619	0.99946	0.99667	
FCN2	–	–	–	–	–	–	–	–

Notable numbers are written in bold letters.

Reference

Kitamura, R., Ishii, K., Maeda, I., Kozaki, T., Iwabuchi, K., and Saito, T. (2016) Evaluation of bacterial communities by bacteriome analysis targeting 16S rRNA genes and quantitative analysis of ammonia monooxygenase gene in different types of compost. *J Biosci Bioeng* **121**: 57–65.
